# Functional *PstI/RsaI* Polymorphism in *CYP2E1* Is Associated with the Development, Progression and Poor Outcome of Gastric Cancer

**DOI:** 10.1371/journal.pone.0044478

**Published:** 2012-09-05

**Authors:** Jin Feng, Xiaolin Pan, Junbo Yu, Zheng Chen, Hao Xu, Wael El-Rifai, Guoxin Zhang, Zekuan Xu

**Affiliations:** 1 Department of General Surgery, The First Affiliated Hospital of Nanjing Medical University, Nanjing, China; 2 Department of Gastroenterology, The First Affiliated Hospital of Nanjing Medical University, Nanjing, China; 3 Department of Surgery, Cancer Biology, and Vanderbilt-Ingram Cancer Center, Vanderbilt University Medical Center, Nashville, Tennessee, United States of America; University of Aberdeen, United Kingdom

## Abstract

**Background:**

Cytochrome P450 2E1 (CYP2E1), an ethanol-inducible enzyme, has been shown to metabolically activate various carcinogens, which is critical for the development and progression of cancers. It has demonstrated that *CYP2E1* polymorphisms alter the transcriptional activity of the gene. However, studies on the association between *CYP2E1* polymorphisms (*Pst*I*/Rsa*I or *Dra*I) and gastric cancer have reported conflicting results. Thus, the aim of the present study was to investigate whether *CYP2E1* polymorphisms is associated with the development and progression of gastric cancer and its prognosis in Chinese patients.

**Methods:**

A case-control study was conducted in which *CYP2E1 Pst*I*/Rsa*I and *Dra*I polymorphisms were analyzed in 510 Chinese patients with gastric cancer and 510 age- and sex- matched healthy controls by PCR-RFLP. Odds ratios were estimated by multivariate logistic regression, and the lifetime was calculated by Kaplan-Meier survival curves. In addition, a meta-analysis was also conducted to verify the findings.

**Results:**

For *CYP2E1 Pst*I*/Rsa*I polymorphism, C2C2 homozygotes (OR = 2.15; CI: 1.18–3.94) and C2 carriers (OR = 1.48; CI: 1.13–1.96) were associated with an increased risk of gastric cancer when compared with C1C1 homozygotes. Both C1C2 and C2C2 genotypes were associated with advanced stage, but not the grade of gastric cancer. Moreover, C2C2 genotype was identified as an independent marker of poor overall survival for gastric cancer. However, there was not any significant association between *CYP2E1 Dra*I polymorphism and the risk of gastric cancer. In the meta-analysis, pooled data from 13 studies confirmed that the *CYP2E1 Pst*I*/Rsa*I polymorphism was associated with a significantly increased risk of gastric cancer.

**Conclusion:**

*CYP2E1 Pst*I*/Rsa*I polymorphism is associated with increased risk of development, progression and poor prognosis of gastric cancer in Chinese patients. Pooled data from 13 studies, mainly in Asian countries, are in agreement with our findings.

## Background

Gastric cancer, one of the most common cancers and the second leading cause of cancer death worldwide, remains an important public health problem. Studies have shown that 989,600 new gastric cancer cases occurred in 2008 and that 738,000 patients die annually of this disease [1]–[2]. There is a considerable geographic variation in the gastric cancer incidence, with higher rates in Asia and some parts of South America. Gastric cancer is one of the most prevalent malignant tumors in China, accounting for 38% of worldwide cases every year [3]–[4]. Although it is well known that environmental factors, dietary habits, and *Helicobacter* pylori infection are associated with the risk of gastric cancer, the host genetic factor is also believed to be important in gastric carcinogenesis [5]. Genetic susceptibilities could be explained, in part, by single nucleotide polymorphisms (SNPs) of susceptible genes [6]–[7]. Therefore, determination and understanding of genetic and molecular factors involved in gastric cancer development and prognosis may help identify novel genetic biomarkers and highlight potential avenues of investigation for targeted therapies.

*Cytochrome P450 2E1* (*CYP2E1*), which is located on chromosome 10q26.3, is an 11.7 kb gene consisting of 9 exons and 8 introns, and encoding a 493 amino acid protein [8]. CYP2E1 is an ethanol-inducible enzyme that metabolically activates various carcinogens, such as benzene, vinyl chloride and N-nitrosamines [9]–[10]. N-nitrosamines are present in tobacco smoke, and activation of N-nitrosamines has been linked to the development of various cancers, including gastric cancer [11]–[12]. Functional *CYP2E1* gene polymorphisms that alter the transcriptional activity of the gene and thus its substances such as N-nitrosamines would influence the susceptibility of cancers. Two genetic polymorphisms in the 5′-flank region (identified by *Rsa*I is −1053C>T (rs2031920) and *Pst*I is −1293G>C (rs3813867), respectively), which are in close linkage disequilibrium, have been reported to alter transcriptional activity of the gene [13]–[14]. The individuals with predominant homozygous allele (C1/C1), the heterozygous allele (C1/C2) and the rare homozygous allele (C2/C2) of *Pst*I*/Rsa*I polymorphism are named the wild-type homozygote, the heterozygote and the rare homozygote, respectively [15]–[17]. Another important polymorphism detectable with *Dra*I in intron 6 is T7632A (rs6413432), a mutation of T to A, which is reported to may alter transcription of the *CYP2E1* gene [13].

Over the last two decades, several studies have explored the association of the *CYP2E1* polymorphism with the risk of lung cancer [18], oral cancer [19], and pancreatic cancer [20]. Recently, a few studies on the association between the *CYP2E1* polymorphism and gastric cancer have also been published, but those studies have yielded contradictory results [21]–[33]. Moreover, there has been no report on the association between *CYP2E1* polymorphism and survival of patients with gastric cancer. Therefore, the aim of this study was to investigate whether *CYP2E1* polymorphism is associated with the development and progression of gastric cancer and its prognosis in Chinese patients. In addition, we also carried out a meta-analysis of selected high quality studies published between 1990 and 2011, in order to reveal more precise association between *CYP2E1* polymorphism and gastric cancer.

## Materials and Methods

### Study Population

The study included 510 patients who were admitted for gastric cancer treatment to the First Affiliated Hospital of Nanjing Medical University between May 2006 and September 2008 and 510 age- and sex-matched healthy controls. All subjects were unrelated ethnic Han Chinese and residents in Jiangsu Province. All cases were newly diagnosed and histologically confirmed without previous chemotherapy or radiotherapy. The pathological stage of gastric cancer was classified according to the tumor-lymph node-metastasis (TNM) classification system into stage I (T1–T2N0M0), stage II (T1–T2N1M0 or T3N0M0), stage III (T3N1M0, T1–T3N2M0, TanyN3M0, or T4NanyM0), or stage IV (TanyNanyM1) [34]. Tumor grade was grouped into low (well differentiated), intermediate (moderately differentiated), or high grade (poor differentiated) according to the World Health Organization (WHO) grade classification [35]. The healthy controls were recruited from individuals living in the same residential areas who took part in routine medical examination at the same hospital withnormal findings during the examination and were age- (±5 years) and sex-matched to the cases.

The study was approved by the Ethics Committee of the First Affiliated Hospital of Nanjing Medical University, and the number of the document was 2008(1101). Written informed consent was obtained from all subjects.

### DNA Extraction and Genotyping of *CYP2E1*

Whole blood was collected into EDTA-coated tubes and centrifuged for 15 min, and the buffy coat layer was isolated. Genomic DNA was extracted from 200 mL of buffy coat using a Qiagen QIAamp DNA Blood Mini kit (Qiagen Inc.,Valencia, CA). Polymerase chain reaction-restriction fragment length polymorphism (PCR-RFLP) was used for gene analysis. The primer structure and restriction enzyme are shown in Table 1. Genotyping was performed without knowledge of the case/control status. The gel images were read independently by two research assistants. If a consensus was not reached on the tested genotypes, then the genotyping was repeated independently until a consensus was reached. To validate the RFLP method, 100 (50 from cases and 50 from controls) samples were selected randomly for direct sequencing with an ABI PRISM Dye Terminator sequencing Kit (Applied Biosystems, Foster City, Calif) with the samples loaded onto an ABI 3700 sequencer. The concurrence rate of these two methods was 99%.

**Table 1:** Gene loci, restriction enzymes and primer sequences.

Gene	Location	Restriction enzyme	primer sequence
CYP2E1	5′-flanking	*PstI*	5′-CCA GTC GAG TCT ACA TTG TCA-3′
			5′-TTC ATT CTG TCT TCT AAC TGG-3′
CYP2E1	Intron 6	*DraI*	5′-TCG TCA GTT CCT GAA AG C AGG-3′
			5′-GAG CTC TGA TGC AAG TAT CGC-3′

### Statistical Analysis

Hardy-Weinberg equilibrium of alleles was assessed by chi-square test. Comparison of age between cases and controls was assessed by the Mann-Whitney U test. The difference in the distribution of genotypes between cases and controls was determined using chi-square test, and the association between the CYP2E1 polymorphisms and gastric cancer risk was estimated by odds ratio (OR) with the 95% confidence interval (CI). Logistic regression was used to control for selected potential confounders (sex, age, and smoking habit) and to estimate crude and adjusted OR and 95% CI. Cumulative overall survival curves were constructed using the method of Kaplan-Meier and the difference was evaluated by the log-rank test. All data analyses were done using SPSS software (version 11.0, Chicago, IL, USA). A *P* value of <0.05 was considered statistically significant.

### Meta-analysis

The electronic databases PubMed, Embase and Web of Science, were searched for studies eligible for inclusion in the present meta-analysis, using the terms: “CYP2E1”, “P4502E1”, “polymorphism(s)”, “gastric” and “cancer or carcinoma or tumor or neoplasm”. An upper date limit of December 5, 2011 was applied, while a lower date limit was 1990. All published English language papers with full text matching the eligible criteria were retrieved. The citations in identified articles and in review articles were also examined. When the same patient population was included in more than one publication, only the most recent or most complete one was included in the meta-analysis. Inclusion criteria included: (a), case-control study on the association between the *CYP2E1 Rsa*I*/Pst*I or *Dra*I polymorphism and gastric cancer; (b), English publication; (c), sufficient published data available for estimating an OR with 95% CI; and (d), the genotypes of the controls consistent with the Hardy-Weinberg equilibrium distribution. Information was carefully and independently extracted from all eligible publications by two of the authors according to the inclusion criteria listed above. Disagreement was resolved by discussion between the two authors. If the two authors did not reach a consensus, a third author was consulted and a final decision was made by the majority of the votes. The following data were collected from each study: first author’s name, publication date, ethnicity, study design, pathological types of gastric cancer, the total numbers of cases and controls, and information on *CYP2E1* (*Pst*I*/Rsa*I and/or *Dra*I) polymorphisms. Then we used the Review Manager 4.2 software **(**The Cochrane Collaboration, Oxford, United Kingdom) for meta-analysis.

## Results

### Characteristics of Study Population

The demographic and pathological characteristics of patients and controls are listed in Table 2. There were no significant differences in demographic data between cases and controls. However, 51.6% of cases were smokers whereas the rate was 44.1% for the controls (OR = 1.35, 95% CI: 1.05–1.73, *P* = 0.017). Of the patients with gastric cancer, 41 (8.0%), 181 (35.5%), 160 (31.8%) and 25 (4.9%) had stages I, II, III, and IV tumors, respectively, and 56 (11.0%), 116 (22.7%) and 231 (45.3%) had low-, intermediate-, and high-grade tumors, respectively.

**Table 2:** Demographic and pathological characteristics of patients and controls.

	Cases (n = 510)		Control (n = 510)		
Variable	No.	%	No.	%	*P* a value
Sex					0.402
Men	321	62.9	308	60.4	
Women	189	37.1	202	39.6	
Age (y)					0.133
<58	240	47.1	264	51.8	
≥58	270	52.9	246	48.2	
Smoking status					0.017
Smoker	263	51.6	225	44.1	
Nonsmoker	247	48.4	285	55.9	
Stage at diagnosis					
I	41	8.0			
II	181	35.5			
III	162	31.8			
IV	25	4.9			
Unknow	101	19.8			
Grade at diagnose					
Low	56	11.0			
Intermediate	116	22.7			
High	231	45.3			
Unknow	107	21.0			

a Two-side chi-square test.

### Association between *CYP2E1* Polymorphisms and Gastric Cancer Risk

The genotype frequencies of the polymorphisms in the controls were consistent with the Hardy-Weinberg equilibrium distribution (*P* = 0.055 for *Pst*I, and *P* = 0.056 for *Dra*I). Table 3 shows the frequency distribution of *CYP2E1 Pst*I or *Dra*I genotypes and the estimated ORs (95% CI) for gastric cancer. In *CYP2E1 Pst*I restriction analysis, there was a significant difference in the distribution of genotypes between the case and control groups. Individuals with the C1C2 or C2C2 genotype had a significantly elevated risk of developing gastric cancer compared with the C1C1 homozygotes, with an adjusted OR (95% CI) of 1.35 (1.01–1.80) and 2.15 (1.18–3.94), respectively. Moreover, C2 carriers (C1C2 or C2C2) had an adjusted OR (95% CI) of 1.48 (1.13–1.96), compared with the C1C1 homozygotes. However, in *CYP2E1 Dra*I restriction analysis, there was no significant difference between the case and control group in the distribution of genotypes. The adjusted OR (95% CI) for TA, AA, and A carriers were 0.76 (0.58–1.01), 1.34 (0.83–2.17), and 0.85 (0.65–1.10), respectively, compared with the TT homozygotes. When the *CYP2E1 Pst*I and *Dra*I genotypes were analyzed together, the individuals with C2C2/AA, had a significantly elevated risk of gastric cancer, with an adjusted OR (95% CI) of 2.66 (1.27–5.57), compared with the C1C1/TT genotype (Table 3).

**Table 3:** Associations between CYP2E1 polymorphisms and gastric cancer risk.

Polymorphism of *CYP2E1*	Case		Control		Crude OR (95% CI)	Adjusted^a^ OR (95% CI)
	No.	%	No.	%		
*Pst*I genotypes						
C1C1	348	68.2	374	73.3	1.00	1.00
C1C2	128	25.1	119	23.3	1.31 (0.98–1.74)	1.35 (1.01–1.80)
C2C2	34	6.7	17	3.3	2.21 (1.21–4.03)	2.15 (1.18–3.94)
C1C2+C2C2	162	31.8	136	26.7	1.42 (1.09–1.86)	1.49 (1.13–1.96)
*Dra*I genotypes						
TT	334	65.5	318	62.3	1.00	1.00
TA	131	25.7	160	31.4	0.78 (0.59–1.03)	0.76 (0.58–1.01)
AA	45	8.8	32	6.3	1.33 (0.83–2.16)	1.34 (0.83–2.17)
TA+AA	176	34.5	192	37.6	0.86 (0.66–1.11)	0.85 (0.65–1.10)
*Pst*I and *Dra*I genotypes						
C1C1/TT	212	41.6	233	45.7	1.00	1.00
C1C2/TA	83	16.3	77	15.1	1.20 (0.83–1.72)	1.13 (0.79–1.63)
C2C2/AA	26	5.1	11	2.2	2.62 (1.26–5.44)	2.66 (1.27–5.57)

a Adjusted for sex, age and smoking habit.

### Association between *CYP2E1* Polymorphisms and Gastric Cancer Disease Status

In *CYP2E1 Pst*I restriction analysis, both C1C2 and C2C2 genotypes were associated with advanced stage, but not the grade of gastric cancer (Table 4). The frequencies of C1C2 genotype were 21.9%, 24.9%, 31.5%, and 44.0% in stages I, II, II and IV, respectively, whereas the frequencies of C2C2 genotype were 4.9%, 4.4%, 11.1%, and 24.0%, respectively. C2C2 genotype was associated with the advanced stage of gastric cancer, among all of the three subgroup analyses (*i.e.* III *vs.* I; III+ IV *vs.* I; III+ IV *vs.* I+ II), and the adjusted ORs (95% CI) were 5.17 (1.05–25.54), 4.80 (1.03–22.45), and 4.38 (1.92–9.97), respectively, compared with the C1C1 homozygotes. In addition, C1C2 genotype was associated with the advanced stage of gastric cancer only in the subgroup analysis comparing stages III+ IV with stages I+ II) (adjusted OR = 1.89; CI: 1.18–3.03), compared with the C1C1 homozygotes. However, no association between CYP2E1 *Rsa*I polymorphism and gastric cancer grade was detected (Table 4).

**Table 4:** Association with CYP2E1 *Rsa*I*/Pst*I polymorphism and progression of gastric cancer.

	Total	C1C1		C1C2		C2C2	
	No.	No.	%	No.	%	No.	%
Tumor stage							
I	41	30	73.2	9	21.9	2	4.9
II	181	128	70.7	45	24.9	8	4.4
III	162	93	57.4	51	31.5	18	11.1
IV	25	8	32.0	11	44.0	6	24.0
Adjusted^a^ OR (95% CI)							
III *vs.* I		1.00		2.29 (0.94–5.58)		5.17 (1.05–25.54)	
III+ IV *vs.* I		1.00		2.30 (0.98–5.38)		4.80 (1.03–22.45)	
III+ IV *vs.* I+ II		1.00		1.89 (1.18–3.03)		4.38 (1.92–9.97)	
Tumor grade							
Low	56	39	69.6	13	23.2	4	7.1
Intermediate	116	79	68.1	20	17.2	17	14.7
High	231	152	65.8	67	20.3	12	5.2
Adjusted^a^ OR (95% CI)							
Intermediate *vs.* Low		1.00		0.59 (0.26–1.35)		1.88 (0.58–6.14)	
High *vs.* Low		1.00		0.50 (0.20–1.26)		0.65 (0.19–2.24)	

a Adjusted for sex, age and smoking habit.

In *CYP2E1 Dra*I restriction analysis, no significant association was observed with either gastric cancer stage or grade (Table 5).

**Table 5:** Association with CYP2E1 *Dra*I polymorphism and progression of gastric cancer.

	Total	TT		TA		AA	
	No.	No.	%	No.	%	No.	%
Tumor stage							
I	41	25	61.0	12	29.3	4	9.7
II	181	122	67.4	38	21.0	21	11.6
III	162	98	60.5	52	32.1	12	7.4
IV	25	15	60.0	7	28	3	12.0
Adjusted^a^ OR (95% CI)							
III *vs.* I		1.00		1.00 (0.45–2.20)		0.90 (0.25–3.17)	
III+ IV *vs.* I		1.00		0.98 (0.45–2.14)		0.93 (0.28–3.11)	
III+ IV *vs.* I+ II		1.00		1.37 (0.86–2.19)		0.75 (0.37–1.52)	
Tumor grade							
Low	56	39	69.6	11	19.7	6	10.7
Intermediate	116	75	64.7	31	26.7	10	8.6
High	231	171	74.0	34	14.7	26	11.3
Adjusted^a^ OR (95% CI)							
Intermediate *vs.* Low		1.00		1.39 (0.62–3.09)		1.02 (0.33–3.12)	
High *vs.* Low		1.00		0.71 (0.33–1.54)		1.08 (0.41–2.81)	

a Adjusted for sex, age and smoking habit.

### Association between *CYP2E1* Polymorphisms and Gastric Cancer Survival

**Figure 1:**
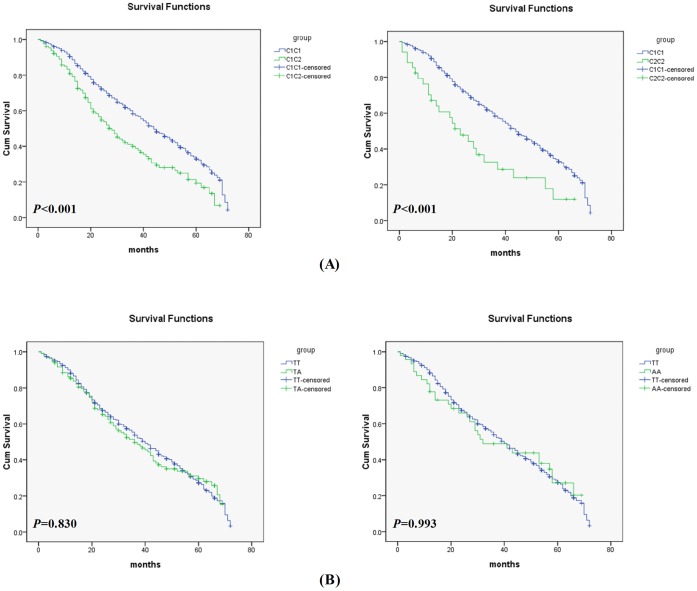
Kaplan-Meier survival curves for gastric cancer patients with *CYP2E1 Pst*I/*Rsa*I (A) and *DraI* (B) polymorphisms. (A), C1C2 or C2C2 genotype had a markedly poor overall survival, compared with C1C1 genotype (*P<*0.001); (B), The survival was not significantly associated with the *CYP2E1 Dra*I polymorphism.

Overall, patients with gastric cancer were followed up for a median (range) of 39 (3–72) months. Kaplan-Meier survival curves (Figure 1A) and log-rank test show that *CYP2E1 Pst*I polymorphism was associated with the poor overall survival of gastric cancer. C1C2 or C2C2 genotype had a markedly poor overall survival, compared with the C1C1 genotype (*P<*0.001). The median estimated cumulative survival was significantly lower in C1C2 or C2C2 carriers (28 months; 95% CI: 22.1–33.9 months, or 23months; 95% CI: 14.0–32.0 months, respectively), compared with C1C1 carriers (44 months; 95% CI, 38.6–49.4 months). However, the survival was not significantly associated with the *CYP2E1 Dra*I polymorphism (Figure 1B).

### Meta-Analysis on the Association between *CYP2E1* Polymorphisms and Gastric Cancer Risk

**Figure 2:**
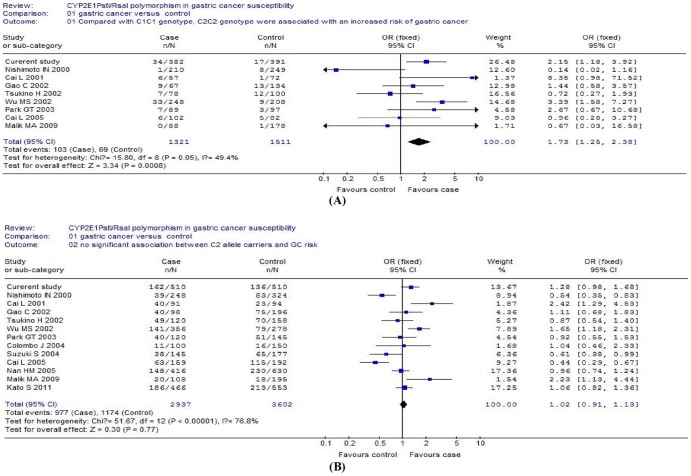
Forest plots on the association of *CYP2E1 Pst*I/*Rsa*I polymorphism with gastric cancer risk. (A), *CYP2E1 Pst*I/*Rsa*I C2C2 genotype and gastric cancer risk (fixed-effect model); (B), *CYP2E1 Pst*I/*Rsa*I C2 allele carriers and gastric cancer risk (random-effect model).

A total of 13 publications met the inclusion criteria [21]–[33]. Of these studies, one study [33] was excluded because the same data was available in a later publication [27]. With the pooled data from those previous studies and our current investigation, this meta-analysis included 2937 cases and 3602 controls. The characteristics of these studies are provided in Table 6. There was a statistically significant association between C2C2 genotype (OR = 1.73; 95% CI: 1.26–2.38; *P* = 0.0008) and gastric cancer risk (Figure 2A). However, there was no significant association between C2 carriers and gastric cancer risk. The pooled ORs for C2 carriers were 1.01 (95% CI: 0.80–1.28; *P* = 0.93), compared with the homozygous wild-type genotype (C1/C1) (Figure 2B). Prior to the present study, there were only two previous studies evaluating the association between *CYP2E1 Dra*I polymorphism and gastric cancer risk [21], [29]. Because the samples of these studies were too small to generate a sufficient power, we did not conduct the meta-analysis on this polymorphism. Nevertheless, all the three studies reported that there was no significant association between *Dra*I polymorphism and gastric cancer risk.

**Table 6:** Characteristics of gastric cancer case-control studies included in meta-analysis on the association between *CYP2E1* polymorphisms and gastric cancer.

Study			Year	Population	Case			Control		
*Pst*I restriction analysis					C1C1	C1C2	C2C2	C1C1	C1C2	C2C2
Park et al		2003		Koreans	80	33	7	94	48	3
Cai et al		2001		Chinese	58	27	6	71	22	1
Nishimoto et al		2000		Brazilian	209	38	1	241	75	8
Tsukino et al		2002		Japanese	71	42	7	88	58	12
Gao et al		2002		Chinese	58	31	9	121	62	13
Colombo et al		2004		Brazilian	89	11	0	134	16	0
Nan et al		2005		Korean	268	148^a^		400	230^a^	
Suzuki et al		2004		Japanese	107	38^a^		112	65^a^	
Wu et al		2002		Chinese	215	108	33	199	70	9
Cai et al		2005		Chinese	96	57	6	77	110	5
Malik et al		2009		Indian	88	20	0	177	17	1
Kato et al		2011		Japanese	280	186^a^		340	213^a^	
Current study				Chinese	348	128	34	374	119	17
*Dra*I restriction analysis					TT	TA	AA	TT	TA	AA
Park et al	2003			Koreans	35	7	78	45	8	85
Wu et al	2002			Chinese	195	120	41	158	100	20
Current study				Chinese	334	131	45	348	121	41

a C1C2+C2C2.

## Discussion

In the present case-control study, for *CYP2E1 Pst*I polymorphism, we observed that both C2 carriers and C2C2 genotypes were significantly associated with gastric cancer risk and poor clinical prognosis. However, we did not found any significant association between *CYP2E1 Dra*I polymorphism and both gastric cancer risk and clinical prognosis. In addition, our meta-analysis also confirmed that the *CYP2E1 Pst*I polymorphism, but not *Dra*I, was associated significantly with the risk of gastric cancer, which provided further evidence indicating an association between this functional polymorphism and gastric cancer susceptibility.

Gastric cancer is a multistep process in which genetic and environmental factors interact in the development of cancer. Interindividual genetic differences in susceptibility to chemical carcinogens are among the most important host factors in human cancer [36]–[38]. It has been proposed that various host factors affect susceptibility to cancer, even following the same exposure to environmental carcinogenic factors [39]–[41]. Of special interest is *CYP2E1* whose polymorphisms are related to substantial interindividual variation in metabolizing carcinogens and cancer risks [42]–[44]. Our results indicating the association between the *CYP2E1* polymorphism and the risk of gastric cancer are biologically plausible. CYP2E1 catalyzes oxidation and DNA adduct formation of some low-molecular carcinogens of gastric cancer [45]–[46]. It has been revealed that the *Pst*I and *Rsa*I restriction sites are in the transcription-regulation region of *CYP2E1* [14]. A tenfold increase in gene expression of the homozygous C2/C2 genotype of *CYP2E1* using *Pst*I or *Rsa*I digestion has been reported [14]. In contrast, the polymorphism detected by *Dra*I digestion of *CYP2E1* is located in intron 6, and no functional significance of this polymorphism exists. This may explain why the *Pst*I/*Rsa*I, rather than *Dra*I, polymorphism of *CYP2E1* conferred a greater risk of gastric cancer in this study. Our meta-analysis provided further evidence indicating an association between *CYP2E1 Pst*I*/Rsa*I polymorphism and gastric cancer susceptibility. It is agreed with the previous meta-analysis in 2007 [47], which reported that CYP2E1 *Pst*I/*Rsa*I polymorphism may be a risk factor for gastric cancer in Asians.

Another interesting finding in the present study was the association of the *CYP2E1 Pst*I*/Rsa*I polymorphism with gastric cancer stage, which may mirror the substantial role of this polymorphism in the progression. We also reported, for the first time, that this polymorphism affected gastric cancer survival. Recently, studies have demonstrated that CYP2E1 plays an important role in tumor progression, and may be used as a prognostic indicator. For instance, Vaclavikova *et al.* observed that increased CYP2E1 expression was associated with an invasive breast cancer, and suggested its potential role as a breast cancer prognosis marker [48]. Tsunedomi *et al.* also found that the expression of CYP2E1 was associated with the progression of hepatitis C virus-associated hepatocellular carcinoma [49]. In addition, CYP2E1 positivity is closely correlated with a poor survival of patients with non-small cell lung carcinoma, and the expression of CYP2E1 in bronchial epithelium has a prognostic potential [50]. Conversely, an animal study demonstrated that blockade of cytochrome P450 significantly reduced capillary formation and tumor size in glial tumors formed by injection of rat glioma 2 (RG2) cells, and also resulted in an increased animal survival time [51]. Furthermore, epidemiologic studies have also indicated that the *CYP2E1 Pst*I*/Rsa*I polymorphism is associated with cancer progression and prognosis [52], [53]. *CYP2E1* C2C2 genotype is significantly associated with advanced clinical stages, and also associated with tumor recurrence, since it is important for determining the parameters associated with tumor progression and poor outcomes in patients with head and neck squamous cell carcinoma [52]. Haque AK *et al.* observed that CYP2E1 wild-type allele was significantly associated with better survival of non-small cell lung carcinoma and the expression of p53 [53]. However, it remains unclear whether *CYP2E1 Pst*I*/Rsa*I polymorphism is associated with the differentiation of cancer. In our study, we found there was no significant association with the differentiation of gastric cancer.

There were a few limitations in the present study. First, data on the cancer stage and differentiation status were unknown for a few patients, which may bring some bias to the results indicating the association between the polymorphism and cancer status. Second, although we carried out a meta-analysis, in order to further confirm our results, only published studies were included in it. This may have limited the power of the pooled results.

In conclusion, *CYP2E1 Pst*I*/Rsa*I polymorphism is associated with development and progression of gastric cancer and poor prognosis of patients with gastric cancer. However, there was no significant association between *CYP2E1 Dra*I polymorphism and the risk of gastric cancer. Future investigation in this area should aim to elucidate the underlying mechanisms between *CYP2E1 Pst*I*/Rsa*I polymorphism and gastric cancer.
